# Mitochondrial Bioenergetics in Brain Following Ozone Exposure in Rats Maintained on Coconut, Fish and Olive Oil-Rich Diets

**DOI:** 10.3390/ijms20246303

**Published:** 2019-12-13

**Authors:** Matthew C. Valdez, Danielle Freeborn, Joseph M. Valdez, Andrew F.M. Johnstone, Samantha J. Snow, Alan H. Tennant, Urmila P. Kodavanti, Prasada Rao S. Kodavanti

**Affiliations:** 1 Neurological and Endocrine Toxicology Branch of the Public Health and Integrated Toxicology Divison, Center for Public Health and Environmental Assessment, Office of Research and Development, U.S. Environmental Protection Agency, Research Triangle Park, NC 27711, USA; valdez.matthew@epa.gov (M.C.V.); freeborn.danielle@epa.gov (D.F.); joevaldez08@gmail.com (J.M.V.); 2 Oak Ridge Institute for Science and Education, U.S. Department of Energy, Oak Ridge, TN 37831, USA; 3 Clinical Research Branch of the Public Health and Integrated Toxicology Divison, Center for Public Health and Environmental Assessment, Office of Research and Development, U.S. Environmental Protection Agency, Research Triangle Park, NC 27711, USA; johnstone.andrew@epa.gov; 4 Cardiopulmonary and Immunotoxicology Branch of the Public Health and Integrated Toxicology Divison, Center for Public Health and Environmental Assessment, Office of Research and Development, U.S. Environmental Protection Agency, Research Triangle Park, NC 27711, USA; samanthajsnow@gmail.com (S.J.S.); kodavanti.urmila@epa.gov (U.P.K.); 5 ICF Consulting, Durham, NC 27713, USA; 6 Rapid Assay Development Branch of the Biomolecular and Computational Toxicology Divison, Center for Computational Toxicology and Exposure, Office of Research and Development, U.S. Environmental Protection Agency, Research Triangle Park, NC 27711, USA; tennant.alan@epa.gov

**Keywords:** mitochondria, ozone, high-fat diet, neurotoxicity, bioenergetics, astrocytes, microglia, oxidative stress, fish oil, olive oil, coconut oil

## Abstract

Dietary supplementation with omega-3 and omega-6 fatty acids offer cardioprotection against air pollution, but these protections have not been established in the brain. We tested whether diets rich in omega-3 or -6 fatty acids offered neuroprotective benefits, by measuring mitochondrial complex enzyme I, II and IV activities and oxidative stress measures in the frontal cortex, cerebellum, hypothalamus, and hippocampus of male rats that were fed either a normal diet, or a diet enriched with fish oil olive oil, or coconut oil followed by exposure to either filtered air or ozone (0.8 ppm) for 4 h/day for 2 days. Results show that mitochondrial complex I enzyme activity was significantly decreased in the cerebellum, hypothalamus and hippocampus by diets. Complex II enzyme activity was significantly lower in frontal cortex and cerebellum of rats maintained on all test diets. Complex IV enzyme activity was significantly lower in the frontal cortex, hypothalamus and hippocampus of animals maintained on fish oil. Ozone exposure decreased complex I and II activity in the cerebellum of rats maintained on the normal diet, an effect blocked by diet treatments. While diet and ozone have no apparent influence on endogenous reactive oxygen species production, they do affect antioxidant levels in the brain. Fish oil was the only diet that ozone exposure did not alter. Microglial morphology and GFAP immunoreactivity were assessed across diet groups; results indicated that fish oil consistently decreased reactive microglia in the hypothalamus and hippocampus. These results indicate that acute ozone exposure alters mitochondrial bioenergetics in brain and co-treatment with omega-6 and omega-3 fatty acids alleviate some adverse effects within the brain.

## 1. Introduction

The health benefits of dietary supplementation with the essential fatty acids (FA) omega-6 and omega -3 have been extensively investigated in regards to their cardiovascular [1,2,3], neurobehavioral [4,5,6], metabolic [7,8,9] and inflammatory effects [10,11,12,13]. Omega-6 fatty acids are polyunsaturated fatty acids (PUFA) found in vegetable oils, nuts and seeds [3]. They are most prevalent in Western diets, where sources such as refined cereal grains are most common [3]. When consumed in moderation and in substitute for saturated fats normally found in meats and dairy products, omega-6 fatty acids can be quite beneficial [2]. Omega-3 fatty acids are generally found in oily fish, lean meat, and green leafy vegetables, and tend to be uncommon in Western diets despite many reports in recent years of their various roles in healthier lifestyles and disease risk reduction [4]. It is conventionally recommended that a 1:1 ratio of omega-6:omega-3 fatty acids is an ideal diet [14]. These health benefits extend to the brain as well. While these PUFAs are incorporated into nearly all lipid bilayers throughout the body, it is noteworthy that the dry weight of the brain is roughly 60% fatty acids, thus highlighting a significant target of accumulating dietary PUFA [15]. PUFAs, specifically omega-3, have been shown to reduce lipid peroxidation and the generation of reactive oxygen species (ROS) while increasing activities of several crucial mitochondrial-based antioxidants in rat brain microvascular endothelial cells [16]. Omega-3 diet supplementation is also protective against propionic-acid-induced neurotoxicity by mitigating many of the effects on neurotransmitter, proinflammatory and proapoptotic markers in vivo [17].

Mitochondria are central regulators of metabolic energy homeostasis and their dysfunction play a key role in neurodegenerative disorders and chemical-induced neurotoxicity [18]. The brain, despite accounting for only 2% of the average body mass, consumes 20% of total resting metabolic energy, most of which is consumed by neurons [19]. Structure and location of neurons lead to different metabolic requirements across the various regions of the brain [20]. To supply energy, mitochondria are responsible for the production of adenosine triphosphate (ATP) via the electron transport chain (ETC) which is comprised of complex enzymes I-IV coupled to ATP-synthase, or Complex V. Complex I, NADH:ubiquinone oxidoreductase, is one of two possible sites where electrons may enter the ETC and is also the main site of the production of ROS [21]. Complex II, succinate dehydrogenase (SDH), reduces ubiquinone to ubiquinol and is the second complex where electrons may enter the ETC. Complex IV, cytochrome c oxidase, transfers electrons from ferro cytochrome c to oxygen, converting it to water and contributes to the electrochemical gradient used for ATP synthesis. Mitochondria are a major source of ROS, as a byproduct of oxidative metabolism, which are typically kept at bay by antioxidants. However, malfunctions in complex enzymes can cause excessive ROS accumulation, leading to oxidative damage [22]. Environmental pollutants such as O_3_, are known to induce oxidative stress (OS) [23]. 

In addition to the intrinsic cellular responses that are activated in neurons to abate excessive ROS production, neighboring glial cells also play a role in mitigating the harmful effects of oxidative stress. Astrocytes, which are typically thought of as facilitators of neurotransmission, also contain large quantities of antioxidants which they can use to contest excessive ROS [24]. For example, reactive astrocytes can mount an antioxidant defense for neurons by the uptake of oxidized vitamin C, converting it to ascorbate (antioxidant) and then releasing ascorbate into the extracellular space neighboring neurons [25]. Other glial cells, namely microglia, are the resident macrophages of the brain. As microglia become activated, they switch from a surveillance and supportive role to a phenotype in which migration, cytokine production, and phagocytosis occur. This role shift is accompanied by a morphological shift from a ramified appearance, with numerous processes and a small cell body, to an amoeboid phenotype, characterized by fewer, shorter processes and a larger cell body [26]. This cascade of events has been implicated in the etiology of major depressive disorders [27]. 

Our previous studies with O_3_ have implicated a role for the brain in mediating a wide array of metabolic and pulmonary inflammatory effects [28,29,30]. A recent report from our group [31], which utilized this same cohort of animals as the present study, found that supplementation of omega-3- and omega-6-containing-dietary oils protected animals from the vasoconstriction response to O_3,_ as well as mitigated the increase in markers of lung injury during O_3_ exposure. However, this paradigm also resulted in pulmonary accumulation of macrophages and inhibition of genes involved in lipid transport. Amongst all dietary supplemented oils, omega-3-containing fish oil had the most influential effect on basal cardiopulmonary and metabolic parameters as well as the responsiveness of these systems to the air pollutant, O_3_. We also found that dietary supplementation with fish oil decreased circulating microRNAs, some of which originated from the brain, and were indicative of an alleviation of tissue-specific distresses or toxicity [32]. Therefore, we hypothesized that dietary supplementation with essential fatty acids might influence the effects of O_3_ on mitochondrial bioenergetics and glial responsiveness. Rats maintained on coconut oil (CO; high in saturated fatty acids), olive oil (OO; high in monosaturated fatty acids), fish oil (FO; high in PUFA) and a normal laboratory diet (ND) were exposed to O_3_ acutely, followed by examination of Complex I, II, and IV in four different brain sections; frontal cortex, cerebellum, hypothalamus, and hippocampus. We also measured ROS production and antioxidant homeostasis in the same regions in order to understand the role of OS. Since diet seemed to elicit greater effects on the mitochondrial bioenergetics, we additionally examined glial morphology in the hypothalamus and hippocampus across diet groups. 

## 2. Materials and Methods 

### 2.1. Animals 

All procedures related to care and handling of animals were performed in accordance with regulations stipulated by the American Association for Accreditation of Laboratory Animal Care (AAALAC) and all experiments were approved by the Animal Care and Use Committee of the National Health and Environmental Effects Research Laboratory at the U.S. Environmental Protection Agency (LAPR # 17-12-002; Dec. 2014–Dec. 2017). Male Wistar Kyoto (WKY) rats were obtained from Charles River Laboratories (Raleigh, NC, USA) at 4 weeks of age and housed two per cage filled with beta chip bedding and enrichment material of crinkled paper. Temperature was maintained at 23 ± 1 °C and relative humidity at 50% ± 10% under a 12 h light/dark cycle (lights on from 06:00 to 18:00).

### 2.2. Dietary Interventions

Diets were employed as described in our previous report [31]. Briefly, animals were fed ad libitum, starting at 4 wks of age, for 8 weeks until O_3_ exposure. They were fed either the normal diet (Rodent Chow 5001, Ralston Purina Laboratories, St. Louis, MO, USA), or a diet supplemented with 6% by weight coconut oil (Teklad Custom Research Diets #TD.140728, Harlan Laboratories, Inc., Indianapolis, IN USA), fish oil (supplemented with Menhaden fish oil—Teklad Custom Research Diets #TD.140729, Harlan Laboratories, Inc.), or olive oil (Teklad Custom Research Diets #TD.140727, Harlan Laboratories, Inc.). Diets were refrigerated throughout the experimentation period and feeding cups were replaced every 2–3 days to minimize oxidation. The three test diets (TD) kept protein, carbohydrates, and fat percentages consistent; only the composition of the fat varied across treatments. See adapted Table 1 [31] for more details on diet ingredients. 

### 2.3. Ozone Exposure

Rats were exposed to filtered air or 0.8 ppm O_3_ for 4 h/day for 2 consecutive days, as previously described [31]. Briefly, O_3_ was generated from oxygen by a silent arc discharge generator (OREC, Phoenix, AZ, USA), and its entry into the Rochester style “Hinners” chambers was controlled by mass flow controllers. Within each chamber, the concentration of O_3_ was continuously recorded by photometric O_3_ analyzers (API Model 400, Teledyne Instruments; San Diego, CA, USA) and maintained within ± 0.01 ppm of the targeted concentration. O_3_ had no effect on environmental variables; including air temperature (23.89 ± 1.78 °C) and relative humidity (49.84% ± 0.30%). During exposure, the O_3_ concentration was monitored consistently; the actual mean chamber concentration was at 0.8045 ± 0.0027 ppm. 

### 2.4. Preparation of Brain Tissue for Enzyme Assays 

Immediately after the second O_3_ exposure, animals were transported to the necropsy room where they were euthanized with an overdose of Fatal Plus (Henry Schein, Melville, NY USA), decapitated, and brains immediately dissected from the skull. Half of the brains were fixed in 4% paraformaldehyde (PFA) for immunohistochemistry, and the other half was dissected on ice to obtain frontal cortex (FC), cerebellum (CB), hippocampus (HIP), and hypothalamus (HYP) and quick frozen on dry ice. All tissue samples were stored at −80 °C until tissue extraction for assessment of mitochondrial complex activities as described below.

Tissue from each brain region was weighed and varying volumes of phosphate buffered saline (PBS, pH 7.3) were added to attain a tissue concentration of approximately 162 mg/mL in order to achieve the necessary 5.5 mg/mL of extract protein required by each assay kit. Tissue was homogenized using a Polytron (Omni International TH, Kennesaw, GA USA) in glass borosilicate tubes (Kimble, Millville, NJ USA) and transferred to a 2.0 mL Eppendorf tube. Ten percent Lauryl maltoside (LM) was added and placed on ice for 30 min. Samples were then centrifuged at 12,000× *g* for 20 min at 4 °C; the resulting supernatant was collected, and the pellet discarded. Supernatant was aliquoted into separate tubes for bicinchoninic acid protein (BCA), Complex I, II, and IV assays, frozen in liquid nitrogen, and stored at −80 °C until analysis. 

For OS markers including ROS production, antioxidant homeostasis, brain tissues were weighed, homogenized with a polytron in 20 mM Tris-HCl buffer (pH 7.4) at 50 mg/mL, and centrifuged at 8000 *g* for 20 min. The supernatants were assayed for the selected OS measures.

### 2.5. Mitochondrial Complex Assays (I, II, IV) 

Enzyme-linked immunosorbent assay kits for mitochondrial Complex I, II, and IV enzymes (Abcam, Cambridge, MA, USA: #AB109721, #AB109908, and #AB109911, respectively) were used to determine enzyme activities in each brain region. Briefly, Complex I activity was quantified by measuring the oxidation of NADH to NAD^+^ and simultaneous reduction of dye, which increases absorbance at 450 nm. The mitochondrial Complex II assay kit catalyzes electron transfer from succinate to the electron carrier ubiquinone. The production of ubiquinone is coupled to reduction of diclorophenolindophenol dye causing it to become colorless and decrease in absorbance at 600 nm. Mitochondrial Complex IV is quantified by measuring the oxidation of reduced cytochrome c, which yields a decrease in absorbance at 550 nm.

Assays were run according to kit instructions, with sample preparation differing as described above for tissue extraction. Absorbance was determined on 96-well plates run on a SpectraMax M5 spectrophotometer running SoftMax ProV5 software (Molecular Devices, San Jose, CA USA). The reaction rates (V_max_) were calculated from the most linear portion of the output curve. All values were standardized by expressing them as activity/mg protein as determined by BCA (Thermo Scientific, Rockford, IL, USA).

### 2.6. Markers of ROS Production

NADH-ubiquinone reductase (UBIQ-RD) was selected as a marker of ROS production as it plays a critical role in several neurodegenerative diseases in which OS is a potential responsible pathway. UBIQ-RD activity was assayed following the method of Cormier et al. [33] where the enzyme catalyzes the oxidation of NADH^+^ H^+^ to NAD^+^, with the ultimate reduction of ubiquinone to ubiquinol. The rate of UBIQ-RD activity was measured as a rotenone-sensitive rate of NADH oxidation at 37 °C and 340 nm.

### 2.7. Markers of Cellular Antioxidant Homeostasis

Total antioxidant status (TAS) was measured using a kit from RANDOX Laboratories (Crumlin, Co., Antrim, UK). ABTS^®^ (2,20-Azino-di-[3-ethylbenzthiazoline sulphonate]) was incubated with a peroxidase (metmyoglobin) and H_2_O_2_ to produce the free radical cation ABTS^®+^. This has a relatively stable blue–green color, which is measured at 600 nm. Antioxidants in the sample cause suppression of this color production in proportion to their concentration [34]. 

γ-Glutamylcysteine synthetase (γ -GCS) activity was determined from the rate of formation of ADP (assumed to be equal to the rate of oxidation of NADH) calculated from the change in absorbance at 340 nm [35]. The above-mentioned colorimetric assays were adapted for use on the KONLAB clinical chemistry analyzer (Thermo Clinical LabSystems, Espoo, Finland).

### 2.8. Immunohistochemistry

The right hemisphere of each brain sample was fixed in 4% PFA for 48 h then stored in 30% sucrose. Two cuts were made perpendicular to the ventral surface of the brain at the level of the optic chiasm and the midbrain, and the resulting brain blocks were mounted in optimal cutting temperature (O.C.T.) compound (Tissue-Tek, Torrance, CA). Coronal sections (50 μm) of the HYP and dorsal HIP were collected with a cryostat (Leica CM1850, Nußloch, Germany) into labeled 24-well plates filled with deOlmos solution [36] and stored at −20 °C. Free-floating brain slices were washed once for 10 min with tris-buffered saline containing 0.0025% Tween-20 (TBS-T) and then incubated for 2 h with a blocking solution (TBS-T containing 10% normal donkey serum, 10% normal goat serum, 0.3% Triton X-100). Blocked sections were incubated overnight at 4 °C on an orbital shaker with two primary antibodies, a polyclonal rabbit anti-ionized calcium-binding adaptor molecule-1 (Iba1, 1:500) antibody (#019-19741; Wako, Richmond, VA, USA) and mouse monoclonal glial fibrillary acidic protein (GFAP, 1:100) antibody (#MA5-12023; Invitrogen, Carlsbad, CA, USA). 24 h later, slices were washed three times in TBS-T for 10 min each wash, incubated for 1.5 h with a mixture of Alexa-488 conjugated anti-mouse antibody (1:200, #A-21121; Invitrogen, Carlsbad, CA) and Alexa-568 conjugated anti-rabbit antibody (1:200, #A-11011; Invitrogen, Carlsbad, CA, USA), washed three times in TBS-T, and incubated for 20 min in 5 μM/mL 4’,6-diamidino-2-phenylindole, dilactate solution (DAPI # D3571; Invitrogen, Carlsbad, CA, USA). ProLong-Diamond antifade (P36961; Life Technologies, Carlsbad, CA, USA) was used to mount the sections on slides. 

A widefield microscope was used to scan and reorder the sections rostral to caudal according to their determined bregma level as defined in the rat brain atlas [37]. Forty micrometer image stacks from the CA1 region of the HYP (bregma -2.00 mm) and dorsal HIP (bregma -3.30 mm) were captured with a Nikon Eclipse Ti A1 confocal microscope fitted with a Plan Apochromat VC 20X objective (Nikon Instruments Inc., Melville, NY USA) under identical settings. Image stacks were analyzed using NIS-Elements (version 4.2, Nikon Instruments Inc., Melville, NY USA). A two-dimensional maximum intensity projection image (MIP) was generated from each channel of each image stack. To assess the extent of GFAP labeling in samples, a binary mask was created using an intensity threshold. Areas exceeding the threshold were deemed positive for GFAP and a sum of that area was calculated for each sample. To assess reactive Iba1 containing cells among all Iba1 labeled cells, an analysis macro written with NIS-Elements used a combination of intensity thresholding and morphometric restrictions to identify and segment labeled cells from the Iba1 channel of the MIP. For each brain region (HYP, HIP), three consecutive image stacks were taken from identical locations from a total of 3 animals/group (*n* = 3 animals). 

### 2.9. Statistical Analysis 

Statistical analyses were performed using RStudio [38]. Raw data were organized with the dplyr package [39]. Normality and heteroscedasticity were determined with the Shapiro–Wilk test and Levene’s tests via *fBasics* [40] and *car* [41] packages, respectively. When subsets of data did not satisfy these assumptions (Complex I in CB, Complex IV in HYP, and Complex II in all regions), the data was log-transformed. 

Two-way analysis of variance was used to analyze each complex assay (Complex I, II, and IV), within each region of interest (FC, HYP, HIP, and CB). Significant effects and/or interactions were followed with two forms of post-hoc tests looking at main effect differences of diet and within-diet group differences of O_3_ exposure. To analyze average effects of diet, a Dunnett’s many-to-one multiple comparison test was performed using the trt.vs.ctrl contrast argument of *emmeans* package [42] (denoted using #). To analyze the effects of O_3_ within each diet, we performed pairwise comparisons using the pairwise contrast argument of *emmeans* (denoted using *).

Nested one-way analysis of variance was used to analyze immunohistochemistry data. Sections from each animal were nested under diet group. Multiple comparisons were performed with a Dunnett’s many-to-one multiple comparison test. All figures were generated using the *ggplot2* [43] and *cowplot* [44] packages. 

## 3. Results

We sought to investigate the effect of dietary oil supplementation with varying ratios of omega-6 and omega-3 on energy homeostasis and neuroinflammation across various brain regions. We additionally wanted to assess if dietary oil supplementation could garner protection from air pollution in the brain. We addressed these questions with two approaches (1) mitochondrial bioenergetics, (2) OS measures, and (3) glia reactivity. We reasoned that since mitochondrial bioenergetics play a key role in multiple basic cellular processes and that dietary oils have been shown to affect mitochondrial respiration [45,46,47], that changes in complex enzyme activities would be a likely outcome. 

### 3.1. Mitochondrial Bioenergetics

**Figure 1:**
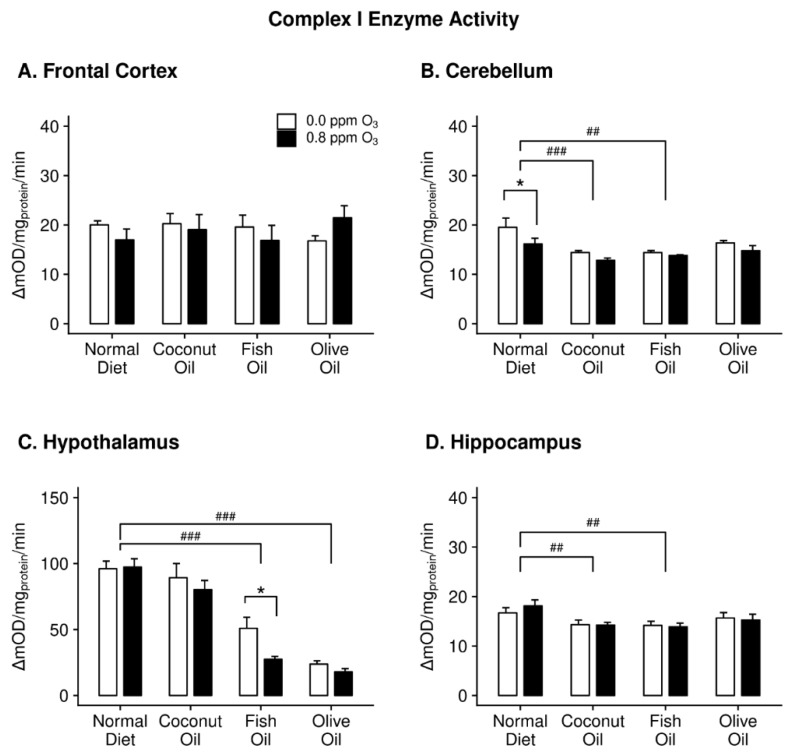
Complex I enzyme activity in the frontal cortex (panel **A**), cerebellum (panel **B**), hypothalamus (panel **C**), and hippocampus (panel **D**) of rats maintained on different diets (normal diet, coconut oil diet, fish oil diet, and olive oil diet) followed by either 0 or 0.8 ppm O_3_ exposure for 4 h/day for two days. Post hoc test results: ## Significantly different from normal diet at *p* < 0.01; ### Significantly different from normal diet at *p* < 0.001; * Significantly different from 0 ppm O_3_ at *p* < 0.05. Data plotted as means ± SEM, *n* = 5.

We measured enzyme activities of mitochondrial complex I, II and IV in the FC, CB, HYP and HIP of rats maintained on diets rich in saturated FA (CO), monounsaturated FA (OO), polyunsaturated FA (FO) and normal diets (ND). Two-way ANOVA indicated a significant effect of diet on complex I activity in the CB (F_3,32_ = 8.078, *p* = 0.0004), HYP (F_3,32_ = 65.066, *p* = 1.032 × 10^−13^) and HIP (F_3,32_ = 4.992, *p* = 0.006) (Figure 1B–D). In both CB and HIP, the CO and FO supplementation decreased the activity of complex I enzyme compared to ND fed animals (Figure 1B,D). CO had almost no effect on complex I in the HYP, however, like in CB and HIP, FO decreased complex I activity in this region along with OO. 

**Figure 2:**
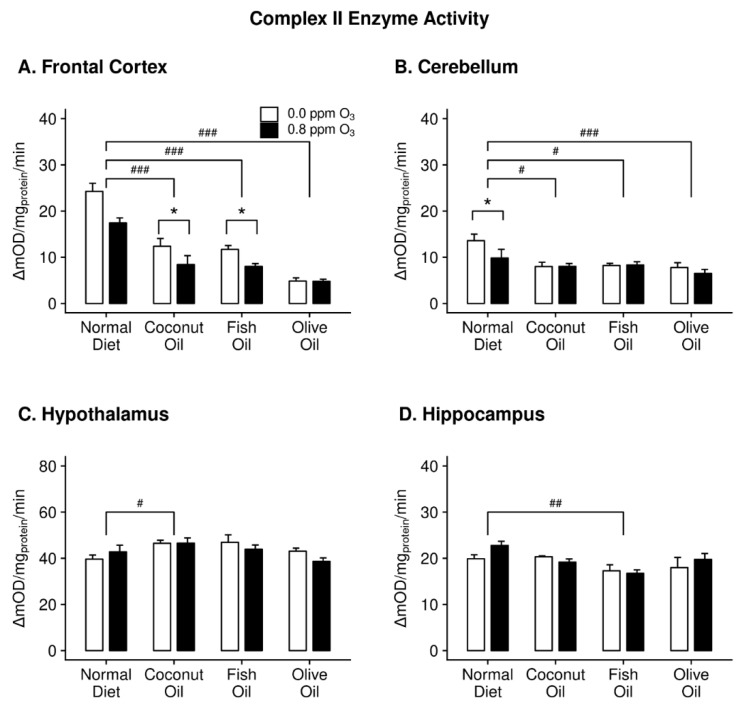
Complex II enzyme activity in the frontal cortex (panel **A**), cerebellum (panel **B**), hypothalamus (panel **C**), and hippocampus (panel **D**) of rats maintained on different diets (normal diet, coconut oil diet, fish oil diet, and olive oil diet) followed by either 0 or 0.8 ppm O_3_ exposure for 4 h per day for two days. Post hoc test results: # Significantly different from normal diet at *p* < 0.05; ## Significantly different from normal diet at *p* < 0.01; ### Significantly different from normal diet at *p* < 0.001; * Significantly different from 0 ppm O_3_ at *p* < 0.05. Data plotted as means ± SEM, *n* = 5.

Two-way ANOVA revealed a significant effect of diet on complex II activity (Figure 2) within all brain regions assayed (FC: F_3,32_ = 46.580; *p* = 8.852 × 10^−12^; CB: F_3,32_ = 6.388, *p* = 0.002; HYP: F_3,32_ = 4.136, *p* = 0.014; HIP: F_3,32_ = 5.045, *p* = 0.006). Interestingly, within the FC and CB, all dietary treatments significantly decreased complex II enzyme activity. In the HYP, there was a slight yet significant increase in complex II activity in the CO group, while all other groups remained at ND activity levels. In the HIP, only FO reduced activity of complex II. 

**Figure 3:**
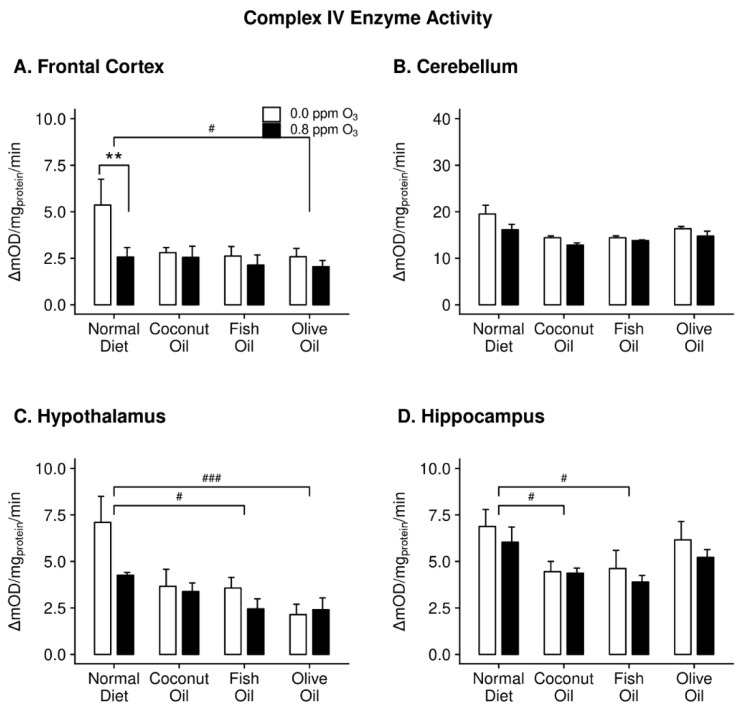
Complex IV enzyme activity in the frontal cortex (panel **A**), cerebellum (panel **B**), hypothalamus (panel **C**), and hippocampus (panel **D**) of rats maintained on different diets (normal diet, coconut oil diet, fish oil diet, and olive oil diet) followed by either 0 or 0.8 ppm O_3_ exposure for 4 h/day for two days. Post hoc test results: # Significantly different from normal diet at *p* < 0.05; ### Significantly different from normal diet at *p* < 0.001; ** Significantly different from 0 ppm O_3_ at *p* < 0.01. Data plotted as means ± SEM, *n* = 5.

Two-way ANOVA demonstrated a nearly significant main effect of diet on complex IV activity in the FC (F_3,32_ = 2.712, *p* = 0.061). All of the diet groups in this region showed complex IV activities that were nearly half of that of the air-exposed ND group. However, post hoc multiple comparisons only showed a significant decrease in complex IV activity in the OO group compared to the ND (Figure 3A). Two-way ANOVA revealed a significant effect of diet on complex IV activity in the HYP (F_3,32_ = 7.1765, *p* = 0.0008) and HIP (F_3,32_ = 4.364, *p* = 0.011). Again, FO caused decreased complex IV activity in both HYP (Figure 3C) and HIP (Figure 3D). Additionally, OO supplementation decreased enzyme activity in the HYP, while CO decreased activity in the HIP. 

We next wanted to determine if the changes in enzyme activity, enumerable above, affected responses to acute O_3_ exposure. In the CB (Figure 1B), we found a significant effect of O_3_ on complex I activity (F_1,32_ = 8.170, *p* = 0.007). A post hoc multiple comparisons test revealed that the only difference between air and O_3_ groups was in ND animals where O_3_ decreased complex I enzyme activity (Figure 1B). There was also a significant effect of O_3_ on complex I activity in the HYP (F_1,32_ = 4.275, *p* = 0.047). Multiple comparisons showed the only difference amongst O_3_ and air groups to be in the FO diet group (Figure 1C). Complex I activity in both FC and HIP were unaffected by O_3_. 

There was a significant effect of O_3_ on complex II activity in the FC (F_1,32_ = 10.445, *p* = 0.003). The differences were determined to be the result of decreased complex II activity in CO- and FO-fed animals exposed to O_3_ (Figure 2A). Within the CB, only the ND animals displayed decreased complex II activity with O_3_ exposure (Figure 2B). O_3_ only affected complex IV activity in the FC (F_1,32_ = 4.686, *p* = 0.039). Post hoc analysis indicated the only O_3_ effect to be a decrease in complex IV activity in the ND (Figure 3A). 

### 3.2. Produciton of Reactive Oxygen Species (ROS)

**Figure 4:**
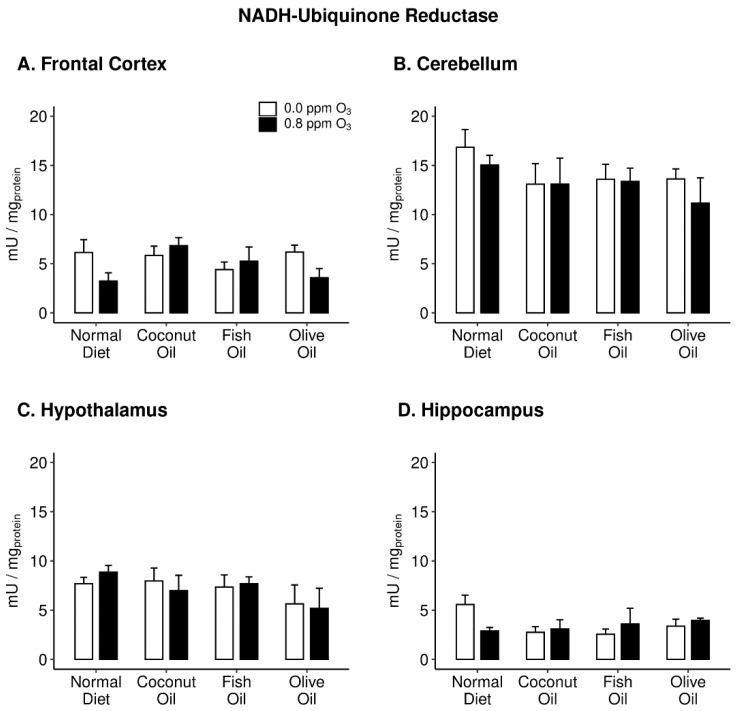
NADH-ubiquinone reductase activity in the frontal cortex (panel **A**), cerebellum (panel **B**), hypothalamus (panel **C**), and hippocampus (panel **D**) of rats maintained on different diets (normal diet, coconut oil diet, fish oil diet, and olive oil diet) followed by either 0 or 0.8 ppm O_3_ exposure for 4 h/day for two days. Data plotted as means ± SEM, *n* = 3–5.

In order to measure ROS, UBIQ-RD (Figure 4) activities were assessed in all brain regions. There were no significant effects of either diet or O_3_ in any of the four brain regions. However, it should be noted that in both FC and HIP, two-way ANOVAs revealed nearly significant interactions of diet and O_3_ (0.08 and 0.07, respectively). 

### 3.3. Total Antioxidant Homeostasis (TAS)

**Figure 5:**
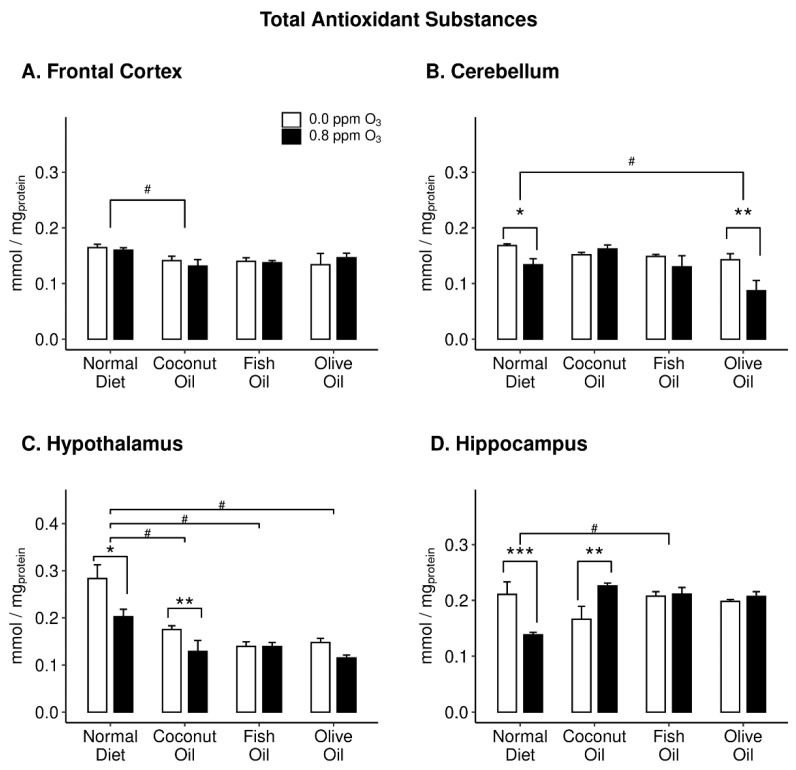
Total antioxidant substances in the frontal cortex (panel **A**), cerebellum (panel **B**), hypothalamus (panel **C**), and hippocampus (panel **D**) of rats maintained on different diets (normal diet, coconut oil diet, fish oil diet, and olive oil diet) followed by either 0 or 0.8 ppm O_3_ exposure for 4 h/day for two days. Post hoc test results: # Significantly different from normal diet at *p* < 0.05; * Significantly different from 0 ppm O_3_ at *p* < 0.01; ** Significantly different from 0 ppm O_3_ at *p* < 0.01; *** Significantly different from 0 ppm O_3_ at *p* < 0.001. Data plotted as means ± SEM, *n* = 5.

Overall, diet and O_3_ had the most pronounced effects on the antioxidant homeostasis. Within the FC (Figure 5A), two-way ANOVA revealed a significant diet effect on TAS (F_3,32_ = 2.959; *p* = 0.050). In this region, the CO decreased TAS compared to ND. Within the HYP (Figure 5C), two-way ANOVA also revealed a significant diet effect on TAS (F_3,32_ = 21.39; *p* = 8.633 × 10^−08^). However, in the HYP, all dietary oil groups had significantly lower TAS compared to ND. Additionally, in the HYP (Figure 5C), two-way ANOVA revealed a significant O_3_ effect on TAS (F_3,32_ = 12.9; *p* = 0.001). In both ND and CO, O_3_ decreased TAS compared to air controls. Perhaps the most intriguing effects found were significant interactions between diet and O_3_ in the CB (F_3,32_ = 2.916; *p* = 0.050; Figure 5B) and HIP (F_3,32_ = 8.67; *p* = 0.0002; Figure 5D). In the CB, O_3_ decreased TAS in the ND and OO groups. In the HIP, O_3_ decreased TAS only in the ND group but increased TAS in CO. 

**Figure 6:**
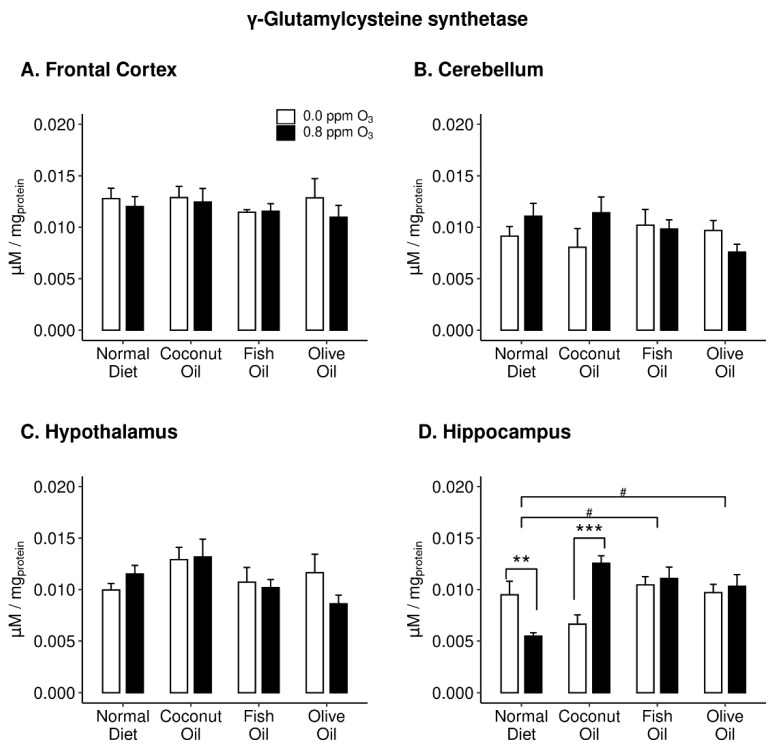
γ-Glutamylcysteine synthetase activity in the frontal cortex (panel **A**), cerebellum (panel **B**), hypothalamus (panel **C**), and hippocampus (panel **D**) of rats maintained on different diets (normal diet, coconut oil diet, fish oil diet, and olive oil diet) followed by either 0 or 0.8 ppm O_3_ exposure for 4 h/day for two days. Post hoc test results: # Significantly different from normal diet at *p* < 0.05 ** Significantly different from 0 ppm O_3_ at *p* < 0.01; *** Significantly different from 0 ppm O_3_ at *p* < 0.001. Data plotted as means ± SEM, *n* = 5.

In addition to TAS, we also measured γ-GCS activity as an indicator of antioxidant status. Two-way ANOVA revealed a significant interaction between diet and O_3_ on γ-GCS within the HIP (F_3,32_ = 9.411; *p* = 0.0001; Figure 6D). Interestingly, the effect of O_3_ on γ-GCS in the HIP mirror those found on TAS in the same region. O_3_ decreased γ-GCS in the ND while increased γ-GCS in CO. 

### 3.4. Glial Morphology

**Figure 7:**
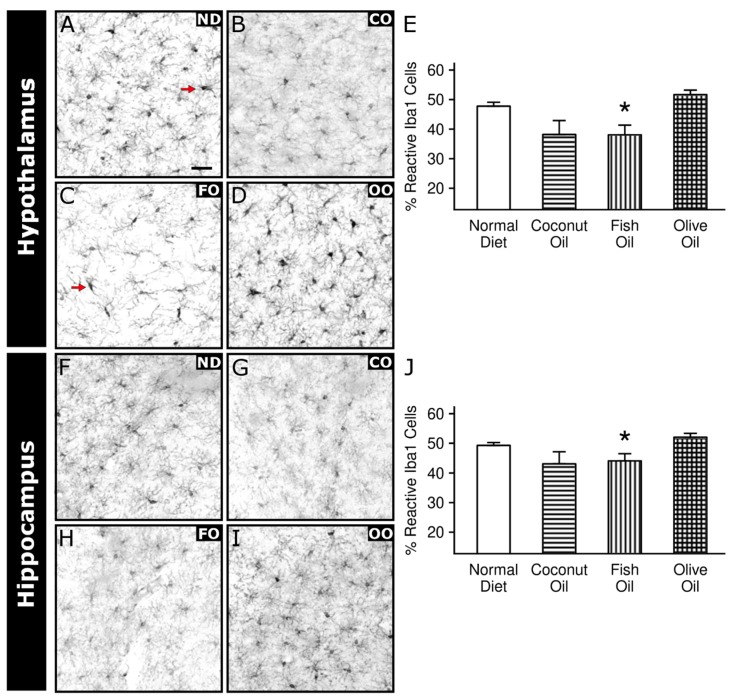
Effects of dietary oil supplementation on microglia. Immunohistochemical analysis of reactive Iba1-containing cells (microglia) from slices of 50 µm were taken from corresponding bregma levels across all animals for hypothalamus and dorsal hippocampus. Representative images of the microglia marker, Iba1, are depicted here by inverting the greyscale image of the corresponding color channel for Iba1 from images taken at 20×. Iba1-containing cells were counted and classified, based on their morphology, as either “resting” or “reactive.” The scoring was automated. The percent of “reactive” cells divided by the total Iba1-positive cells for each image were analyzed within each brain region. Hypothalamus: normal diet (**A**), coconut oil (**B**), fish oil (**C**), olive oil (**D**) quantitative analysis (**E**). Hippocampus: normal diet (**F**), coconut oil (**G**), fish oil (**H**), olive oil (**I**) quantitative analysis (**J**). Post hoc test results: * Significantly different from normal diet at *p* < 0.05. Data plotted as means ± SEM, *n* = 3. Images have been scaled 2× for better visualization in print and, therefore, do not contain the full area of analysis. Scale bar represents 10 µm. Red arrows indicate microglia with representative reactive morphology.

Based on the mitochondrial bioenergetic data, dietary oil supplementation appeared to influence change in homeostasis across brain region more often and to a greater extent than acute O_3_ exposure. Therefore, we next focused our investigation on the influence of dietary oils on neuroinflammation and astrogliosis. Microglia are the resident macrophages of the central nervous system and undergo profound morphological changes when activated. To assess neuroinflammatory response enacted by microglia, we assayed the number of reactive microglia in the HYP and HIP of air exposed animals across all diet groups (Figure 7). Microglial activation was significantly affected by dietary oil in the HYP and the HIP (F_3,49_ = 4.219, *p* = 0.0099 and F_3,67_ = 3.583, *p* = 0.018, respectively). In both cases, post hoc multiple comparisons revealed that FO diet decreased the percentage of reactive cells compared to animals fed a ND (Figure 7E,J). 

**Figure 8:**
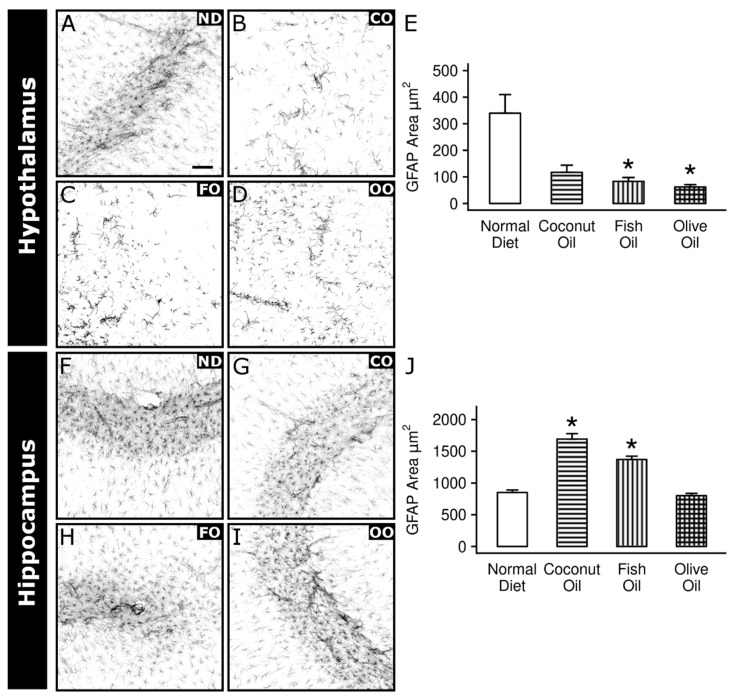
Effects of dietary oil supplementation on astrocytes. Immunohistochemical analysis of astrocytic GFAP coverage from slices of 50 µm were taken from corresponding bregma levels across all animals for hypothalamus and dorsal hippocampus. Representative images of the astrocytic marker, GFAP, are depicted here by inverting the greyscale image of the corresponding color channel for GFAP from images taken at 20×. GFAP immunoreactivity was traced via NIS Elements software and area (µm^2^) data were analyzed within each brain region. Hypothalamus: normal diet (**A**), coconut oil (**B**), fish oil (**C**), olive oil (**D**) quantitative analysis (**E**). Hippocampus: normal diet (**F**), coconut oil (**G**), fish oil (**H**), olive oil (**I**) quantitative analysis (**J**). Scale bar represents 20 µm. Post hoc test results: * Significantly different from normal diet at *p* < 0.05. Data plotted as means ± SEM, *n* = 3.

Likewise, astrocyte area (µm^2^) was significantly affected in the HYP and HIP (F_3,55_ = 3.177, *p* = 0.031 and F_3,38_ = 37.63, *p* = 6.30 × 10^−15^). FO and OO decreased the GFAP area in the HYP (Figure 8E) compared to a ND while CO and OO diets increased the GFAP area in the HIP (Figure 8J). 

## 4. Discussion

The objective of this study was to examine the effects of CO-, FO-, and OO-supplemented diets, relative to ND on neuronal and glia parameters and if these diets afforded protection against acute ozone exposure. A previous report from our group [31], which utilized the same cohort of animals, found that supplementation of different dietary oils impacted the vasoconstriction response to O_3,_ as well as mitigated markers of lung injury while inducing foamy macrophage accumulation_._ One of the most interesting findings from this previous study was also the stark decrease of ubiquitous serum microRNAs in these animals, which may be indicative of tissue-specific or global changes in toxicity. Analysis of the sources of these microRNAs revealed that most were derived from the spleen and brain. This decrease in serum microRNA appeared to be driven primarily from FO supplementation as there were nearly no effects of acute O_3_. Given the role of the brain (i.e., hypothalamic pituitary adrenal axis activation) in mediating the pulmonary damage of acute O_3_ exposure [28,30,48], we sought to investigate changes in both neurons and glial cells across the brain that could help us understand the changes imparted by dietary oil supplementation and if the protective effects described in the vasculature are also present in the brain. 

Few studies describe the effects of chronic low-dose exposure to O_3_ on the brain [23,49,50]. These studies use a dose of 0.25 ppm 4 h/day exposure for a duration of 7–90 days, in contrast to our 0.8 ppm 4 h/day two-day exposure. Our goal in choosing a higher acute dose was to determine the effects of an acute exposure to understand how initial injury and inflammation might be impacted by specific FA-rich diets. Other studies have also used acute doses and found increased lipid peroxidation that increases with dose at a 4 h exposure as low as 0.4 ppm O_3_ in male Wistar rats [51]. Overexpression of vascular endothelial growth factor (VEGF), a factor associated with cellular recovery after brain injury, can be observed after as little as a 3 h exposure to 0.5 ppm O_3_ [52] in brain glial cells. Although damage may be present at higher, acute doses, a more chronic exposure is necessary to demonstrate O_3_-diet-protective effects. In our recent study, we have shown that an acute single ozone exposure induces gene expression changes in the brainstem and HYP, that reflect a generalized response, such as hypoxia, suggesting a potential involvement of mitochondria (Henriquez et al., 2019).

Mitochondria not only play a central role in energy production but ostensibly play critical roles in the initiation and propagation of various signaling cascades [53,54,55]. We found significant effects of both O_3_ and dietary oil supplementation that were brain-region- and complex-enzyme-specific. In nearly every brain region, we found significant effects of diet (various oil supplementations compared to ND). The general trend was that unsaturated oil supplementation decreased complex enzyme activity. It is important to mention how we interpreted the decrease in enzyme activity that we measured in this study. The assays employed here are based on the rate of substrate utilization or product generation as monitored over time by the absorbance of a chemical indicator. In the simplest interpretation, decreased enzyme activity is to be taken as an indication of a decrease in the *amount* of functional enzyme. This then raises the question as to the nature of the *dysfunction*, if any, that is resulting in less enzyme activity. This could be a result of damaged complex enzymes by several mechanisms including oxidative stress [56]. Alternatively, this decrease in enzyme could indicate increases in apoptosis as reported by others [57], as a result of dietary oil supplementation. In this case, the upregulation of apoptosis would effectively decrease the number of mitochondria present in each brain region. Furthermore, it is important when looking at changes in enzymatic activity in different brain regions to consider differing basal levels in these regions, such as with animals fed the ND in this study. Heterogeneity of the brain regions and susceptibility to mitochondrial injury is affected by many factors including number and distribution of mitochondria, enzyme activities and enzyme expression levels. Mitochondria in regions of the same neuron can even differ between dendrites, soma, and axons [20]. Therefore, the heterogeneity of the brain regions should be considered when interpreting these results. 

We did find evidence of neuroprotection of mitochondrial bioenergetics in half of the brain regions analyzed. Within the CB, O_3_ decreased complex I in the ND animals only. CO, FO and OO showed no effect of O_3_ exposure; however, this appeared to be due to the already depressed activities in all these groups. Within the FC, we found a similar trend, complex IV activity was only decreased by O_3_ in the ND group while all other diet groups displayed lower enzyme activities with no effect of O_3_. There were other regions that appeared to follow this trend, but the decreases were not significant. Why diet garnered some level of protection from O_3_ only in these regions and with these enzymes is unclear from the data. 

In all regions expect for CB, we found activity changes in Complex IV when there were corresponding changes in either Complex I and/or II. This coincides with previous results where Complex I and II activity seem to correlate with cytochrome oxidase activity [20]. This would suggest that affecting the input to the ETC through complex I or II would have consequences for the final output as well. Further investigation would be required to ascertain whether this is a chain affect or the complexes are being affected separately. In our study, animals fed a CO-enriched diet have increased complex II enzyme activity compared to ND in the HYP (Figure 2). Pandya et al., 2016 postulated that out of rodents aged 4, 12, and 24 months, a succinate-fueled metabolism was preferred in younger rats and speculated that the immature brain preferentially uses complex II as an entry point to the ETC. In this study, the rats began consumption of test diets at 4 wks of age and were approximately 12 wks of age at time of tissue collection. Their young age and the postulated preference for succinate as an entry point to the ETC at this age could explain the increased Complex II activity in the HYP with animals fed CO. 

We also examined markers of OS, specifically, oxyradical production (stimulation of UBIQ-RD) and antioxidant homeostasis (TAS and γ-GCS). UBIQ-RD is the first complex of the electron transport chain found within the inner membrane of mitochondria and is crucial for ATP production [58], while also playing a role in neurodegenerative diseases [59,60]. There were no effects of diet or O_3_ on oxyradical production across all brain regions.

However, endogenous antioxidant levels were affected in specific brain regions. Antioxidants are a critical tool for cells to combat ROS damage in all tissues, including the brain, and their activity is highly influenced by the cause and region of damage [61]. TAS and γ-GCS were measured in the same brain regions. TAS measures the amount of total antioxidants available within a tissue and allows us to assess the capacity of said tissue to oppose ROS. In all brain regions except FC, O_3_ decreased TAS under ND. Interestingly, there were instances across all brain regions where O_3_ affected TAS except for FO indicating a potential role in buffering TAS levels in the brain. γ-GCS enzyme activity indirectly measures the abundance of glutathione, as it is the rate-limiting step in its production. Glutathione, a potent antioxidant, can prevent damage from ROS and the magnitude of its activity garners insight into the levels of ROS present. Only one region (HIP) showed altered γ-GCS activity. Furthermore, the activity levels corroborate the TAS measures, but only in this region. Recently, it has been demonstrated that HIP requires large pools of glutathione to sustain dendritic integrity [62]. Therefore, it is plausible that glutathione may be the dominate antioxidant in the HIP, thus driving the measure of TAS for that region. 

To further investigate the effect of diet on the brain, we focused on the morphometric changes in glia cells in the most affected brain regions, the HYP and HIP. FO supplementation decreased number of reactive microglia cells in both HYP and HIP. Interestingly, in both brain regions, the general effect of FO throughout all enzyme complexes was a significant decrease in activity. If the decrease in enzyme activity was related to any sort of mitochondrial and cellular damage, a neuroinflammatory response would be likely. Surprisingly, the lack of reactive microglia suggests that no such neuroinflammatory response was present. The lack of neuroinflammation could be due to increases in antioxidant activity attributed to the dietary oils, thereby reducing oxidative damage and thus suppressing signals that would typically activate microglia cells. FO has been shown to decrease age-related dysfunction of mitochondria [63] and lipid peroxidation while at the same time increase antioxidants such as superoxide dismutase in cortex [64]. Upregulation of these protective homeostatic functions may require less intervention by microglia and indicate more efficient mitochondrial bioenergetics. 

The effect of dietary supplemented oils on astrocytes has been less investigated. We demonstrate a profound impact of dietary oil supplementation on astrocytic GFAP. Interestingly, this affect appears to be region-specific as the decreased GFAP response to unsaturated oils in the HYP shifts towards an increase in the HIP. The increase in GFAP suggests an increase in astrocytic activation. Docosahexaenoic acid (DHA) is one of the main omega-3 FA found in FO and has been shown to increase GFAP expression in brains of offspring via the fatty acid-binding protein-7 when exposed in utero [65]. Whether this mechanism persists to influence astrocytes postnatally has not been explored. However, this increase in activated astrocytes has been demonstrated before in response to another type of air pollutant, fine particulate matter (PM_2.5_). In a rodent model of ischemic stroke, PM_2.5_ exacerbated activation of astrocytes and was associated with decreased locomotion [66]. Given the role of astrocytes in combating oxidative stress and maintaining optimal conditions for neighboring neurons, the increase in GFAP coverage in the HIP is indicative of a perturbance, which is partially supported by the decrease in complex enzyme activity in the region. Conversely, the same diet groups decreased astrocytic GFAP in the HYP. It is notable that the HYP is the only region tested in the study that displayed an increase in complex enzyme activity and decrease in microglial activation, which may imply benefit of dietary oil supplementation. Others have shown similar decreases in astrocytic activation using monosaturated oils like OO in culture. Amyloid beta oligomers are hallmark biomarkers in the etiology of Alzheimer’s disease and induce inflammation characterized by interleukin-6 increase and GFAP upregulation. Co-application of cultured astrocytes with oleocanthal, a monosaturated FA found in OO, ameliorates these affects [67].

Neuroinflammation is co-morbid with numerous neurodegenerative diseases such as Alzheimer’s disease [68], Parkinson’s disease [69], Huntington’s disease [70] and amyotrophic lateral sclerosis [71]. Neurodegenerative diseases are categorized by progressive neuronal loss, and increased levels of cytotoxic substances, such as extracellular debris, pro-inflammatory factors, and production of ROS, resulting in OS. These factors can activate and recruit microglia [72]. Therefore, when considering the effect of microglial activation and OS, the consequences on neuronal health must be taken into consideration. For example, our results from the HIP demonstrate that FO decreases reactive microglia while increasing TAS and γ-GCS in O_3_-exposed animals. Although indirect measures, when considered with previous data from our group [31] showing decreased serum microRNA originating from brain, an indicator of celluar damage, we begin to see evidence of neuroprotection from dietary oils in response to air pollution. Evidence correlating air pollution and neurodegenerative diseases has gained interest recently [73,74,75]; however, to role of diet as a mitigating factor has been overlooked. As more data linking air pollution and neurodegenerative diseases comes out, future studies on the of use of practical mitigation strategies, such diet, to combat the progress of the neuronal loss are needed. 

## 5. Conclusions

In conclusion, we found that unsaturated FA supplementation, specifically FO, garnered some defense for specific brain regions from O_3_-induced decreases in complex enzyme activity. However, the effects of O_3_ on mitochondrial bioenergetics are not consistent across enzyme complexes nor across brain region, which suggests that different brain compartments may be more or less susceptible to air pollutants such as O_3_. Also, while diet and O_3_ have no apparent influence on endogenous ROS production, they do affect antioxidant levels in the brain. FO was the only diet that O_3_ exposure did not alter. Moreover, as we found that diet significantly affects the reactivity of both astrocytes and glia cells in animals treated with O_3_, future investigations are warranted to understand how diets rich in unsaturated fatty acids may serve as neuroprotectants. 

## Figures and Tables

**Table 1:** Composition of diets used in the current study.

Content (G/Kg)	Normal (Purina 5001)	Coconut Oil (Td. 140728)	Fish Oil (Td. 140729)	Olive Oil (Td. 140727)
Protein	250.0	177.0	177.0	177.0
Carbohydrate	475.0	600.6	600.6	600.6
Fat	64.0	72.0	72.0	72.0
Fiber	53.0	50.0	50.0	50.0
Saturated Fa	14.8	56.6	18.4	11.8
Monounsaturated Fa	16.2	6.2	16.0	45.7
Polyunsaturated Fa	10.0	7.0	31.3	12.5
Omega 3 Fa (N-3)	3.0	0.8	20.3	1.2
Eicosapentaenoic Acid	UNK	0.0	9.6	0.0
Docosahexaenoic Acid	UNK	0.0	6.5	0.0
Omega 6 Fa (N-6)	10.5	6.2	7.8	11.3
N-6/N-3 Ratio	3.5	7.8	0.4	9.4
Metabolizable Energy (Kcal/Gm)	4.09	3.8	3.8	3.8

FA = Fatty Acids; UNK = unknown; values not routinely measured.
